# Detection of Non-Polio Enteroviruses From 17 Years of Virological Surveillance of Acute Flaccid Paralysis in the Philippines

**DOI:** 10.1002/jmv.23242

**Published:** 2012-02-15

**Authors:** Lea Necitas Apostol, Akira Suzuki, Analisa Bautista, Hazel Galang, Fem Julia Paladin, Naoko Fuji, Socorro Lupisan, Remigio Olveda, Hitoshi Oshitani

**Affiliations:** 1 Department of Virology, Tohoku University Graduate School of MedicineSendai, Japan; 2 Virology Department, Research Institute for Tropical MedicineMuntinlupa, Philippines; 3 Tohoku-RITM Collaborating Research Center for Emerging and Reemerging Infectious DiseasesMuntinlupa, Philippines; 4 World Health Organization-Regional Office for the Western PacificManila, Philippines

**Keywords:** circulation pattern, epidemiology, neutralization test, NPEV rate, polio eradication

## Abstract

Acute flaccid paralysis (AFP) surveillance has been conducted as part of the World Health Organization (WHO) strategy on poliomyelitis eradication. Aside from poliovirus, which is the target pathogen, isolation, and identification of non-polio enteroviruses (NPEVs) is also done by neutralization test using pools of antisera which can only identify limited number of NPEVs. In the Philippines, despite the significant number of isolated NPEVs, no information is available with regard to its occurrence, diversity, and pattern of circulation. In this study, a total of 790 NPEVs isolated from stool samples submitted to the National Reference Laboratory from 1992 to 2008 were analyzed; neutralization test was able to type 55% (442) of the isolates. Of the remaining 356 isolates, which were untyped by using neutralization test, 348 isolates were analyzed further by RT-PCR targeting the VP1 gene. A total of 47 serotypes of NPEV strains were identified using neutralization test and molecular typing, including 28 serotypes of human enterovirus B (HEV-B), 12 serotypes of HEV-A, and 7 of HEV-C. The HEV-B group (625/790; 79%) constituted the largest proportion of isolates, followed by HEV-C (108/790; 13.7%), HEV-A (57/790; 7.2%), and no HEV-D. Coxsackievirus (CV) B, echovirus (E)6, E11, and E13 were the most frequent isolates. E6, E11, E13, E14, E25, E30, E33, CVA20, and CVA24 were considered as endemic strains, some NPEVs recurred and few serotypes existed only for 1–3 years during the study period. Despite some limitations in this study, plural NPEVs with multiple patterns of circulation in the Philippines for 17 years were identified. ***J. Med. Virol. 84:624–631, 2012***. © 2011 Wiley Periodicals, Inc.

## INTRODUCTION

The human enteroviruses (HEVs) continue to represent a significant global public health threat. HEVs are small, non-enveloped RNA virus that is the largest member of the family Picornaviridae. More than 90 antigenically distinct serotypes are known to cause infections in humans and based on the pathogenicity in humans and in experimental animals, enteroviruses were classified previously as polioviruses (PVs), coxsackieviruses A and B (CVA and CVB), echoviruses (E), and the numbered enteroviruses (EV). On the basis of nucleotide and amino acid sequences of the viral protein genes, a new classification was proposed and HEVs have been subgrouped into seven species namely HEV-A (17 serotypes), human enterovirus B (HEV-B) (58 serotypes), HEV-C (16 serotypes and three PVs), and HEV-D (three serotypes) and rhinovirus A, B, and C (http://talk.ictvonline.org and http://www.picornaviridae.com).

Most infections are mild, asymptomatic, or subclinical, thus rendering non-polio enteroviruses (NPEVs) to be less important as human pathogens worthy of sustained investigation compared to other viral infections or diseases of greater perceived public health importance. However, NPEVs can cause a broad spectrum of clinical conditions and may also result in serious or even fatal outcome. Enteroviruses are viruses implicated most commonly in of acute myocarditis, acute hemorrhagic conjunctivitis, hand–foot–mouth disease with neurological complications and aseptic meningitis [Goh et al., 1990; Khalfan et al., 1998; Ho et al., 1999; Zhang et al., 2004]. EV infections among neonates and infants also occur with varying degrees of severity [Piraino et al., 1982; Hawkes and Vaudry, 2005; Jordán et al., 2009]. The PV, the cause of paralytic poliomyelitis, still causes significant disability in some countries such as Nigeria, Pakistan, Afghanistan, and India. Furthermore, several studies provide more evidence that enteroviruses are also the causative or contributory agents to chronic diseases including insulin-dependent diabetes mellitus and dilated cadiomyopathy [Hovi et al., 1996; Lönnrot et al., 2000; Zhang et al., 2004].

One of the key strategies for eradication of poliomyelitis set by World Health Organization (WHO) is laboratory-based detection of PV through acute flaccid paralysis (AFP) surveillance. In line with this WHO strategy, the national AFP surveillance program was established in the Philippines in 1992. Since then, this surveillance system has been the only source of information on EV infection for the country. Laboratory test for EV is conducted for stool specimens from patients with AFP. The last indigenous wild-type polio was detected in 1993 and the Philippines was declared polio-free in 2000. Through the years, poliomyelitis cases have been reduced worldwide and eradicated in some parts of the world, leaving the NPEV as one of the implicated causes of AFP. In the Philippines, NPEVs account for about three-fourths (798/1,089) of the total isolates from 1992 to 2008 in AFP surveillance. Among these NPEVs, almost half were untypable using WHO protocols on enterovirus detection (WHO Poliovirus Laboratory Manual, 2004). Identifying the serotype circulating in the country may help in understanding epidemiology of enterovirus infection and to a larger context of polio eradication, may give the assurance that PV is not overlooked. Laboratory data were analyzed to give an overview of identified NPEV serotypes and their occurrence, diversity, and pattern of circulation by showing the results during a 17-year study period as implicated in AFP in the Philippines.

## METHODS

### Samples

A total of 11,079 stool samples collected from 5, 268 children aged <15 years who were presenting acute flaccid type of paralysis from January 1992 to December 2008 were sent to the WHO National Reference Laboratory for PVs at the Research Institute for Tropical Medicine. Most of the cases had two stool specimens with an interval of 24–48 hr between collections and within 14 days from date of onset of paralysis. Out of these stool samples, 798 NPEVs (7.2%) were isolated during the study period of 17 years, however, only 790 isolates were included for analysis in this study since other eight samples could not be found.

Initially, the neutralization test was carried out on the 798 NPEVs by using the pools of antisera. Serotyping was done on all RD-positive isolates according to the time frame set by WHO that result must be released within 28 days of receipt of the specimens. The method enabled serotype identification of 55% (442) of the NPEV isolates. From review of records, the remaining isolates including the missing eight samples (356) yielded no neutralization pattern and were thus reported as untypable NPEV (uNPEV). Since the present study is not part of the routine surveillance program on the basis of typing all NPEVs, a microneutralization test was not repeated, instead, the remaining 348 uNPEV isolates were analyzed by a molecular method.

### Virus Isolation

All samples were inoculated onto the WHO-recommended cell lines for HEV detection: RD and HEp2C (Cincinnati) cell lines were used from 1992 to 1997 and from 1997 onwards, RD and L20B cells were used. L20B, a genetically engineered mouse cell line, is intended for the specific isolation of PV but recently, it has been reported that it can also propagate some CVA types 4, 8, and 10 [Nadkarni and Deshpande, 2003]. When complete cytopathic effect (CPE) characterized by rounding necrosis was observed, the infected cells were harvested and kept frozen (−20°C) until typing. For samples showing no CPE after 7 days, a re-passage in same cell line was done. In this study, the 790 isolates which were isolated by RD cells according to records of data were investigated.

### Serotype Identification by Neutralization Test

Microneutralization tests with pools of antisera specific for common EV serotypes were used for all the virus isolates obtained, according to WHO standard protocols. Serotype-specific immune antisera for the CVB and some echovirus serotypes which were available only until 2000 and EV pools from the National Institute for Public Health and the Environment, Bilthoven, the Netherlands were used. The EV pools contain reference horse typing antisera against the HEV serotypes isolated most frequently combined as nine antiserum pools: polyclonal antisera against a trivalent pooled polio antiserum (PP pool), a CVB1-6 pool (CP pool), a CVA9, and echoviruses (A-G pool). The pools can only identify 27 out of more than 90 existing HEV serotypes. Briefly, diluted isolates were mixed with equal volumes of the aforementioned antisera pools. Daily microscopic observation was performed to check for the presence of characteristic rounding necrosis CPE. The antiserum that prevented the development of CPE indicated the identity of the virus [WHO, 2004]. The isolates which did not present any neutralization pattern of CPE were then reported as uNPEV.

### RT-PCR on 5′ Untranslated Region (UTR) and Partial Sequencing of the Viral Polyprotein 1 (VP1) Region

Reverse transcriptase-polymerase chain reaction (RT-PCR) on 5′ UTR and partial sequencing of the VP1 capsid region were performed for the 348 isolates which were isolated on RD cells and previously reported as uNPEV. In brief, viral RNA was extracted from culture supernatants using Purelink Viral DNA/RNA kit (Invitrogen, Carlsbad, CA) according to the manufacturer's instruction. Thereafter, complementary DNA was synthesized using extracted RNA with random primers, M-MLV, and RNAse OUT (Invitrogen). The 5′ UTR was amplified and used as screening test for enterovirus and exclusion as described previously [Iturriza-Gómara et al., 2006]. For typing, HEV capsid gene (VP1) sequences were amplified by a previously described PCR protocol using primers 222 and 292, leading to fragments of around 350 bp [Oberste et al., 2003]. In a total volume of 20 µl, 1 µl cDNA was used for PCR, together with 0.8 µl of 10 µM each primer, 2 µl 10× ExTaq Buffer (Takara, Otsu, Japan), 1.6 µl dNTP mix and 1 µl Taq polymerase (Takara), and 13.7 µl H_2_O. Thermocycling was performed in GeneAmp® PCR System 9700 (Applied Biosystems, Foster City, CA) for 50°C for 30 min, 94°C for 3 min followed by 30 cycles at 94°C for 30 sec, 42°C for 30 sec, and 72°C for 30 sec with ramp time of 1 min. The reaction products were analyzed by electrophoresis in a 2% Agarose/0.5 TBE gel stained with ethidium bromide. All VP1 positive samples with the expected amplicon size were subjected to purification using SUPREC PCR (Takara). Purified products were then labeled directly by cycle sequencing reaction with BIG DYE Terminator v.1.1 and were analyzed by ABI 3730 Genetic Analyzer (Applied Biosystems).

### Data Analyses

Automated DNA sequencing of the amplified fragments was carried out on both strands and alignment of the obtained sequences with selected reference serotypes from GenBank was performed using the Clustal W algorithm as implemented in MEGA version 4.0 [Tamura et al., 2007]. Subsequently, the results were analyzed by the BLAST algorithm. In this study, a previously described criteria on VPI sequence identity was adopted [Oberste et al., 2003].

Due to the variation in the total number of specimens received and isolates detected annually, a priori cut-off point was set to define patterns of circulation during the study period: endemic is defined when a serotype was isolated for at least 8 years; cyclic are those serotypes which were isolated for at least 4 years; epochal are those serotypes which occurred in 1–3 years, within the 17-year period.

## RESULTS

### Identification of Isolates

#### Virus isolation and serotyping

Since the nationwide surveillance for AFP was implemented in the Philippines in 1992 up to 2008, 798 NPEVs (7.2%) were isolated during the study period of 17 years. Microneutralization test enabled the serotype identification of 55% (442) of the NPEV isolates, mostly belonging to HEV-B group. The remaining isolates (356) yielded no neutralization pattern and were thus reported as uNPEV.

#### Molecular typing

The viral genomes of 348 samples were positive for the 5′UTR and VP1 regions. The sequences obtained in VP1 were compared with those included in GenBank database and were assigned the serotype of the strain that gave the highest identity score. The sequences revealed their serotypes, when homology in VP1 sequence was at least 75% to prototype strains.

Results of serological and molecular tests were summarized in Table I. Sequence data revealed that 52% of the isolates (181/348) could have been typed by the EV antisera pool. However, CVA3-6, CVA8, CVA10, CVA13-14, CVA16-17, CVA19-21, CVA24, E19, E24, EV71, EV73, EV74, EV76, EV77, EV80, EV90, EV96, EV99, which are not included in antisera pool were identified only through molecular method (167/348). Unexpectedly, PV 1 was detected. Later, it was confirmed by two reference laboratories in Australia and in Japan that it was Sabin-like PV 1.

**Table I:** Summary of Isolates Identified by Serological and Molecular Tests

Isolate	EV pool	VP1 PCR	Total	Isolate	EV pool	VP1 PCR	Total
HEV-A	**1**	**56**	**57**	Echovirus 12	15	(2)	17
Coxsackievirus A4		12	12	Echovirus 21	12	(2)	14
Coxsackievirus A10		9	9	Echovirus 20	8	(3)	11
Coxsackievirus A3	1	7	8	Echovirus 4	10		10
Enterovirus 71		8	8	Echovirus 24		9	9
Coxsackievirus A8		7	7	Echovirus 27	4	(4)	8
Enterovirus 90		4	4	Echovirus 1	2	(6)	8
Coxsackievirus A5		2	2	Echovirus 2	5	(1)	6
Coxsackievirus A6		2	2	Enterovirus 80		4	4
Coxsackievirus A16		2	2	Echovirus 5	2	(1)	3
Coxsackievirus A19		1	1	Echovirus 9	2	(1)	3
Enterovirus 76		1	1	Enterovirus 77		3	3
Coxsackievirus A14		1	1	Enterovirus 73		3	3
HEV-B	**438**	**187 (147)**	**625**	Enterovirus 74		2	2
Coxsackievirus B	132	(10)	142	Echovirus 31		(2)	2
Echovirus 11	41	(34)	75	Coxsackievirus A9	1		1
Echovirus 13	31	(20)	51	HEV-C	**3**	**105 (34)**	**108**
Echovirus 7	32	(9)	41	Coxsackievirus A24^a^	1	36 (23)	37
Echovirus 6	33	(7)	40	Coxsackevirus A20^a^	2	24 (9)	26
Echovirus 30	21	(12)	33	Coxsackievirus A17		10	10
Echovirus 19^a^	1	27 (8)	28	Coxsackievirus A13		10	10
Echovirus 29	21	(5)	26	Enterovirus 96		10	10
Echovirus 3	23	(2)	25	Coxsackievirus A21		7	7
Echovirus 25	19	(3)	22	Enterovirus 99		6	6
Echovirus 14	17	(3)	20	Poliovirus 1		(2)	2
Echovirus 33	6	(12)	18	Total	**442**	**348 (181)**	**790**

Bold numbers represent the total number of isolated and detected non-polio enteroviruses by genogroup, that is, HEV-A.

Numbers in parenthesis could have been typed by serological test.

a aSerotype-specific antisera were available until 2000.

#### Frequency and periodicity of EV serotype among AFP cases

**Fig. 1:**
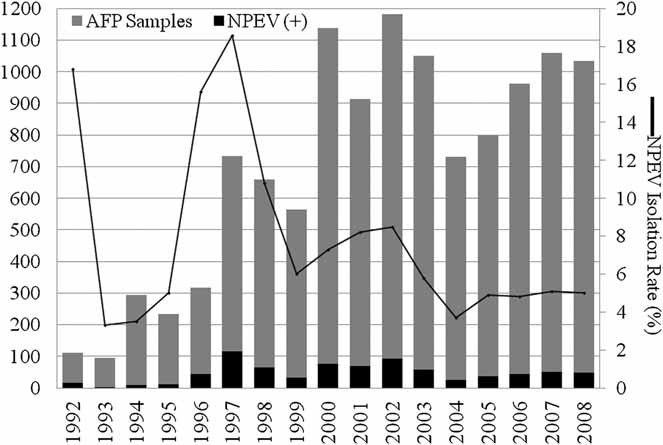
Non-polio enterovirus (NPEV) isolation rate from acute flaccid paralysis (AFP) cases, Philippines, 1992–2008.

During the study period, the NPEV mean isolation rate was 7.8%, with only 4 years (1992, 1996–1998) reaching more than 10% (Fig. 1). Of the 790 samples, 57 belonged to HEV-A (7.2%), 625 to HEV-B (79.0%), and 108 to HEV-C (13.7%). No isolate from the HEV-D species was detected during the span of 17 years. A great proportion of the total NPEV population in the Philippines were species under the HEV-B with CVB group (18%) having the highest number of the total isolates (Table II). No serotype was identified for CVB using the serological test since the EV pool contains only CVB-polyspecific antisera. Serotypes with the highest prevalence varied each year, with CVB, E11, and E13 documented to occur predominantly for 17 years.

**Table II:** Distribution of Non-Polio Enterovirus Serotypes by Year

Serotype	1992	1993	1994	1995	1996	1997	1998	1999	2000	2001	2002	2003	2004	2005	2006	2007	2008	Total
HEV-A																		
CV A4						3					2	3			4			12
CV A10				1						1	2			4	1			9
CV A3									1		4			1		2		8
EV 71									2		4			2				8
CV A8						2					2			1		2		7
EV 90											2	2						4
CV A6											2							2
CV A16																	2	2
CV A5											2							2
CV A19										1								1
CV A14											1							1
EV76																	1	1
Total	***0***	***0***	***0***	***1***	***0***	***5***	***0***	***0***	***3***	***2***	***21***	***5***	***0***	***8***	***5***	***4***	***3***	***57***
HEV-B																		
CV B	8		1		2	24	19	4	12	8	21	5	8	6	6	12	6	142
E11			2	3	7	6	10	3	3	9	4	6		4	8		10	75
E13				1	7	9	4	2	4	6	2	3	2	3		8		51
E6	8	1			3	2		2	6	3	8	1	3		3			40
E30			1		4	6	2		3	3	2	3			2	5	2	33
E25		2		2		2	3			1	4	2			2	2	2	22
E14			1		2	4	4					2	2		1		4	20
E33			1		2			4	1			2		2	3	3		18
E7			2			4			15			5		2	1		12	41
E3				2		16				2		1		2	2			25
E29						4	1		3	14					2		2	26
E19					4				5			3	8			7	1	28
E12					4			6			2	1	2		2			17
E21							2		1	7		1		2		1		14
E20											3	2		4			2	11
E4			2							2	2			2			2	10
E27							2	2		3			1					8
E24						3						5				1		9
E1						2				3		2				1		8
E2							5			1								6
EV 80											1				2		1	4
E5							2				1							3
EV 77											2	1						3
E9				1			1				1							3
EV 73				1					2									3
EV 74									1			1						2
E31						2												2
CV A9											1							1
Total	***16***	***3***	***10***	***10***	***35***	***84***	***55***	***23***	***56***	***62***	***54***	***46***	***26***	***27***	***34***	***40***	***44***	***625***
HEV-C																		
CV A24					3	7	1	3	10		6	2				3	2	37
CV A20					2	6	2		1	2	8	1		2	1	1		26
CV A13						5			1	1	2					1		10
CV A17					2	2			1		2	1				2		10
EV 96					1	2	6					1						10
CV A21									2			2			3			7
EV 99						2		3	1									6
POLIO 1										2								2
Total	***0***	***0***	***0***	***0***	***8***	***24***	***9***	***6***	***16***	***5***	***18***	***7***	***0***	***2***	***4***	***7***	***2***	***108***
Total	**16**	**3**	**10**	**11**	**43**	**113**	**64**	**29**	**75**	**69**	**93**	**58**	**26**	**37**	**43**	**51**	**49**	**790**

Bold italic numbers emphasize the total number of serotypes in each genogroup, that is, HEV-A while numbers in bold denotes the grand total of all serotypes (or genogroups' combined total) in the study.

Color legends: endemic: clear; cyclic: gray; and epochal: dark gray.

E11was the most predominant serotype followed by, in descending order, E13, E7, E6, CVA24, E30, E19, E29, CVA20, and E7, all of which were identified almost annually except E19, E29, E7, and E3, which appeared to be recurrent. Other serotypes (CVA4, CVA10, CVA3, E12, E21, E4, E27, CVA13, CVA17, and EV 96) have the tendency of recurrence at variable time intervals, ranging from 4 to 7 years. Also, epochal viruses, most of which were identified just recently (EV76, EV90, EV80, EV77, EV73, EV74, EV96, and EV99) and rare serotypes (CVA8 and CVA14), have been observed during the study period. Notably, using the criteria for circulation pattern, no HEV-A was identified as endemic. HEV-B circulated predominantly as endemic, cyclic, and epochal; similarly, HEV-C in the Philippines showed three defined circulation patterns but with few isolates.

## DISCUSSION

WHO protocols for enterovirus detection and identification for AFP surveillance are based on virus isolation followed by serotyping the isolated viruses by neutralization test which allows the identification of only 27 enterovirus serotypes [Muir et al., 1998]. About 44% (348/790) of the NPEV isolates were not identified by neutralization test, however almost half of the isolates that were typed by molecular testing (181/348: 52.0%) could have been typed serologically by using pools of antisera. These isolates had been overlooked in serotyping possibly due to “breakthrough” because of high-viral concentration used in neutralization test rendering an inconclusive pattern or antigenic drift. For as long as the L20B, a highly selective cell line for PVs, is negative and that PP pool does not neutralize, these isolates can be reported simply as NPEVs. In the context of AFP surveillance, PVs are the pathogen of interest and detailed testing for identification is not required for NPEVs.

**Fig. 2:**
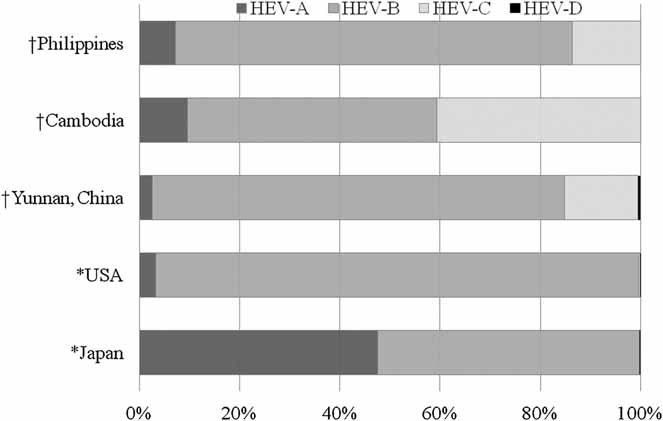
Proportion of enterovirus isolates in tropical (†) and temperate (*) countries Cambodia: [Arita et al., 2005]; Yunnan, China: [Bingjun et al., 2008]; USA: [Khetsuriani et al., 2006]; Japan: [Infectious Disease Surveillance Center, 2000; Infectious Disease Surveillance Center, 2002; Infectious Disease Surveillance Center, 2008].

The incidence of different species of enteroviruses in different countries is shown in Figure 2. The data from temperate countries demonstrate an isolation frequency pattern: HEV-B > HEV-A > HEV-C > HEV-D. In contrast, the data in tropical countries including our present study reveal the higher isolation of HEV-C species than HEV-A (HEV-B > HEV-C > HEV-A > HEV-D) [Arita et al., 2005; Tian et al., 2007]. Evidences on the frequency of recombination among PVs with the HEV-C have been published [Brown et al., 2003; Rousset et al., 2003; Kew et al., 2004; Shimizu et al., 2004; Arita et al., 2005; Rakoto-Andrianarivelo et al., 2007]. Shimizu et al. reported that the circulating vaccine-derived PV type 1 detected in the Philippines contains an unidentified donor strain at the non-structural protein coding region which was considered to be derived from the HEV-C species. The relatively high frequency of HEV-C detected in this study might help in identifying the origin and the molecular mechanism of emergence of cVDPVs in the Philippines. While the data in Figure 2 have come from different settings, the records in tropical countries which only included the data from AFP surveillance may serve as partial picture of the occurrence of NPEVs. Data from temperate countries like Japan and USA are derived from a more comprehensive enterovirus surveillance that includes enterovirus infections from broad clinical categories and the environmental surveys, thus may reflect the overall NPEV isolation frequency [Infectious Disease Surveillance Center, 2000; Infectious Disease Surveillance Center, 2002; Khetsuriani et al., 2006; Infectious Disease Surveillance Center, 2008], whereas published data in tropical countries came from AFP surveillance alone. Additional data is needed to elucidate the actual patterns of circulating enteroviruses in tropical countries. The apparent non-detection of HEV-D can be attributed to the fact that occurrence of EV68 and EV70 maybe limited only in patients with respiratory syndromes [Oberste et al., 2004] and acute hemorrhagic conjunctivitis, respectively [Bern et al., 1992; Bhide et al., 1994; Maitreyi et al., 1999]. Recently, the isolation of the newly proposed member of HEV-D, EV94, was the only reported HEV-D species to have been isolated from an AFP case [Smura et al., 2007].

The results indicated that multiple patterns of circulation of NPEV in the Philippines over 17 years existed and that, plural serotypes co-circulated each year. Circulation of individual serotypes varied from endemic, cyclic, and epochal patterns (Table II). The cyclic emergence (recurrence) of enterovirus serotypes (CVA10, E7, E3, E29, E19, E12, E21, CVA13, and CVA17) may suggest that these serotypes have circulated probably during a period when high population immunity have waned and reintroduction occurs when a new build-up of susceptible individuals take place. The introduction of single serotype at different years and the existence of epochal strains with few isolates are patterns which can be explained by importation and epidemic transmission and not endemic serotypes of the country.

In this study, recently identified EVs have been documented: EV73, EV74, EV76, EV77, EV80, EV90, EV96, and EV99. These viruses along with E19, CVA24, and E24 are not included in the EV antisera pools. It can be noticed that activities of E29 (2001), CVA24 (2000), E7 (2000), and E3 (1997) were marked with high isolations. However, review of records showed that E29 isolations were collected on almost the same week with only 2 days interval (data not shown) in Region 10 of the Philippines. Though the data might suggest that there was a circulation of E29 in a community in 2001, a possible outbreak cannot be dismissed. In addition, HEV-A and HEV-C and PV 1 isolates were missed by serologic test and were identified through molecular method. The polio isolate was confirmed later as Sabin-like by both the regional (Victorian Infectious Diseases Reference Laboratory, Melbourne, Australia) and global (National Institute of Infectious Diseases, Tokyo, Japan) reference laboratories. Record-tracing revealed that the PV detected which was isolated and reported as uNPEV in year 2000 had a negative result on the first inoculation in both RD and L20B cell lines and on re-passage, showing characteristic CPE only in RD but not in L20B. The event prompted the laboratory to proceed on performing the microneutralization test which resulted into no neutralization pattern in EV pools (PP, CP, and A-G pools). After the molecular identification of PV 1 in the present study and before sending to the global reference laboratories for further characterization, verification of the true identity of the PV was done by reinoculating the samples onto RD as well as L20B and by serotyping using monospecific polyclonal PV antisera to types 1, 2, and 3 combined as antiserum pools. Both cell lines, RD and L20B, presented the characteristic CPE and microneutralization test concluded that the sample was indeed, a PV 1. While the polio case was missed, the event points out two aspects: (i) it is a vital component that proper inoculation of samples must be ensured and that vigilance while performing the experiment, i.e., inoculation, must be observed; (ii) it underlines the importance of employing molecular methods to help us interpret trends and patterns and elucidates the partial, if not actual, burden of disease caused by NPEVs and their contribution to the PV surveillance and as the goal of eradication is approaching, to assure that PV cannot be missed.

The isolation of rarely reported enteroviruses like CVA3, CVA8, CVA13, CVA14, CVA17, CVA19, CVA20, CVA21, E12, E29, EV73, EV74, EV76, EV77, EV80, EV90, EV96, and EV99 during the study period was observed. According to epidemiological data on EVs from Europe, USA, Latin America, China, and Japan, these viruses have limited isolation frequency, with only <100 detections worldwide [Infectious Disease Surveillance Center, 2000; Trallero et al., 2000; Infectious Disease Surveillance Center, 2002; Khetsuriani et al., 2006; Tian et al., 2007; Infectious Disease Surveillance Center, 2008]. Nevertheless, two of these reported rarely isolated serotypes provided by the present study, E29 and E12, have relatively high isolation frequency in the Philippines. Moreover, this study reveals isolation of uncommon EVs such as CVA3, CVA8, CVA19, EV 74, EV76, EV 77, EV80, EV 90, and EV 99, which are among the viruses with almost limited detection worldwide except in the Philippines. Whether the occurrence of these rare viruses is just a mere introduction to the country remains unclear, as sequence data in other countries are limited. Furthermore, the clinical spectrum of the rare serotypes is yet to be defined because all specimens were collected only from AFP cases.

NPEVs are common in children, however, its importance in the Philippines has not been well documented even if large outbreaks occurred in its neighboring countries. For example, E11, E13, and E30 are well known among the serotypes of EVs causing aseptic meningitis and along with EV71 which is the causative agent of hand–foot–mouth disease with some severe complications. These outbreaks due to NPEVS were commonly reported in the Western-Pacific countries like Japan, Korea, Taiwan, Malaysia, and China [Kit, 2002; Tian et al., 2007; Baek et al., 2009]. Some of these viruses were identified in this study, but clinical and epidemiological importance in the Philippines is still to be determined since only AFP cases were investigated. Routine virological surveillance for aseptic meningitis and encephalitis as well as HFMD cases is required to clarify their potential importance.

The minimum NPEV rate or sensitivity measurement for PV surveillance set by the Western Pacific Region is greater than 10% [WHO, 1996]. The set rate was achieved only for 4 years (1992, 1996–1998) as shown in Figure 1. The observed declining trend in NPEV rate may be due to a true reduction of NPEV incidence among AFP cases and likewise, field-influenced factors such as collection of stool specimens of more than 14 days from onset date of paralysis and transport time lag from date of send-out to receipt of specimens in the laboratory may have affected the viability of the specimens and subsequently had reduced yield of isolation. However, correlation studies on these factors are warranted. Likewise, the omission of HEp2C which was replaced by L20B cell line in 1997 could have reduced further the NPEV isolation rate [WHO, 2004]. Nevertheless, despite the fact that Hep2C was excluded as one of the recommended cell lines for routine use, the present study had isolated and detected a multitude of viruses, especially the CVB which contributed significantly to the annual NPEV rate. While the sensitivity of RD cells for CVB is reported to be low in previous reports [Schmidt et al., 1975; WHO, 2004], the present study was able to isolate and detect CVB using the RD cell line provided by WHO Collaborating Center in Australia. Whether the characteristic of the RD cells used is different from other previous studies which may have resulted in higher isolation in this study cannot be known. Thus far, the result of this study supports a recent research stating that RD is sensitive to almost all types of enteroviruses, including CVB [Tsao et al., 2010].

This study is subject to a number of limitations. First, detection of EV in line with the AFP surveillance is the only data source where occurrence of EV infections could be traced back. Enterovirus surveillance encompassing all related infections has not taken place in the Philippines. Thus the data may be underrepresented of the overall impact of NPEV infection in the country. Furthermore, overrepresentation of neurotropic NPEV types may probably bias the data if AFP cases will be the only core of identifying NPEVs. Nonetheless, their detection may either reflect their causative role in AFP or the possibility that these enteroviruses just transiently localize the gastrointestinal tract and are being shed in stool specimen. Second, some CVA strains that cannot be propagated in RD and HEp2C may have been under-detected and the growth of other EV types may have been favored [Hosoya et al., 2002]. The L20B cell line is highly selective for PV and only few NPEVs may grow. Until now, no cell line has been identified to isolate all the existing NPEV types.

Based on the nationwide surveillance of AFP-associated infections in the Philippines, this study examined systematically the datasets since 1992–2008 in order to elucidate the proportion, diversity, circulation pattern of NPEVs. In conclusion, although EV detection is only a “part-outcome” of the PV surveillance, different patterns of circulation of plural enterovirus serotypes were detected.
